# IoMT–Blockchain framework for secure and real-time heart disease monitoring using hybrid black-winged optimized spherical structural graph convolutional neural networks

**DOI:** 10.1038/s41598-026-57620-0

**Published:** 2026-06-11

**Authors:** P. Bhuvaneshwari, Mohammad Zubair Khan, Cyril Prasanna Raj, M. S. Shreya Yadav, S. Muthuvel

**Affiliations:** 1 Manipal Institute of Technology Bengaluru, Manipal Academy of Higher Education, Manipal, India; 2 Faculty of Computer and Information Systems, Islamic University of Madinah, 42351 Madinah, Saudi Arabia; 3 Department of Electronics and Communication Engineering, Cambridge Institute of Technology, K R Puram, Bengaluru, 560036 India; 4 Department of Computer Science and Engineering, Academy of Maritime Education and Training (AMET University), Kanathur, Chennai, Tamil Nadu India

**Keywords:** Blockchain, Internet of Medical Things, Heart disease monitoring, Hybrid structural graph attention network, Spherical convolutional neural network, Computational biology and bioinformatics, Engineering, Health care, Mathematics and computing

## Abstract

Cardiovascular diseases are a primary global health concern, requiring continuous monitoring and accurate diagnostic mechanisms for early detection and prevention. The integration of the Internet of Medical Things (IoMT), deep learning, and blockchain technologies has enabled intelligent healthcare systems capable of real-time cardiac assessment and enhanced security in the management of medical information. However, existing heart disease monitoring systems are affected by noisy physiological signals, inefficient feature extraction, limited classification accuracy, and insufficient security in distributed environments, motivating the development of a robust and reliable diagnostic framework with improved security. To address these challenges, this research proposes an IoMT and Blockchain-Based Heart Disease Monitoring System Using Hybrid Black-Winged Spherical Structural Graph Convolution Neural Network (HBW-SSG-CNN). The proposed workflow begins with IoMT-based acquisition of ECG and PCG signals, followed by preprocessing using quasi-cross bilateral filtering (QCBF) to suppress noise while preserving critical cardiac structures. Signal decomposition is performed using Spectral Envelope-Based Adaptive Empirical Fourier Decomposition (SE-AEFD), followed by feature extraction using the Short-Time Quaternion Quadratic Phase Fourier Transform (ST-QQPFT). Feature dimensionality is optimized using the Success-Based Optimization Algorithm (SBOA), and heart disease classification is performed using a Hybrid Structural Graph Attention Network with Spherical Convolutional Neural Network (HS-GAT-SCNN), further enhanced by the Black-Winged Kite Algorithm (BWKA). To enhance data integrity and improve security, an Adaptive Hash Algorithm with Weighted Probability Model (AHA-WPM) is integrated with a Fair Consensus Blockchain for Heterogeneous Miners (FCB-HM). The proposed model is evaluated using publicly available benchmark datasets, including PhysioNet cardiac signal datasets and the Cleveland heart disease dataset. Experimental evaluation demonstrates superior performance with an accuracy of 99.21%, sensitivity of 98.94%, specificity of 99.08%, and F1-score of 99.02%. The results confirm that the proposed framework provides a highly accurate, scalable, and security-enhanced solution for near real-time heart disease monitoring in IoMT-enabled healthcare systems.

## Introduction

The rapid development of digital technologies has dramatically changed modern healthcare systems by enabling continuous monitoring, real-time data sharing, and decision support^1^. The Internet of Medical Things is one such development. It has become a vital paradigm in which medical sensors, wearable devices, and healthcare infrastructure are linked via networked communication technologies^2^. IoMT enables continuous collection of physiological data, which is necessary for early diagnosis, long-term disease management, and individualized healthcare^3^. Wearable medical devices also improve this ecosystem by enabling non-invasive, long-distance health monitoring and reducing reliance on hospital-based care^4^.

Heart disease has been among the most common causes of deaths around the globe and has been placing a significant burden on economies and health sectors^5^. Cardiovascular diseases involve the cardiac rhythm, blood circulation, and myocardial activity, and most of the time cause critical complications like stroke and heart failure^6^. Disease prevalence is highly promoted by risk factors, which are hypertension, smoking, drinking alcohol, obesity, stress, and sedentary lifestyles^7^. Constant observation of cardiac procedures, including electrocardiogram and phonocardiogram recordings, has thus become necessary for promptly detecting and preventing adverse cardiac events^8^.

The vast amount of sensitive health information generated by IoMT devices raises significant concerns about data security, privacy, and integrity during storage and transmission^9^. Traditional centralized systems are susceptible to unauthorized access, data tampering, and single-point failures^10^. Blockchain technology is a cryptographically secured, decentralized framework that can enhance data immutability, traceability, and trust among stakeholders in the healthcare sector^11^. Blockchain can be used to securely share medical data and enhances reliable transmission without third-party integration by leveraging distributed ledgers and secure authentication protocols^12^.

IoMT and blockchain, when combined, form a robust healthcare monitoring architecture that enhances secure information collection, transparent access control, and uncompromised storage^13^. This type of integration is especially useful for monitoring heart disease, where physiological data and clinical observations must be accurate and reliable at all times^14^. High-level analytical algorithms also facilitate meaningful interpretation of biomedical indicators and clinical parameters, thereby improving diagnostic accuracy^15^. Together, IoMT and blockchain technologies create a scalable, secure, and efficient foundation for new heart disease monitoring systems.

### Contribution

- Presents an IoMT-enabled cardiac challenges system that, in real time, receives ECG and PCG heart activity signals from wearable sensors, thus providing continuous, non-invasive observation of heart activity.
- Use of Quasi-Cross Bilateral Filtering (QCBF) to reduce noise and baseline drift and still maintain clinically significant cardiac morphological structures.
- Uses Spectral Envelope-Based Adaptive Empirical Fourier Decomposition (SE-AEFD) to break nonstationary cardiac signals into physiologically significant frequency components.
- Survives discriminative time–frequency-phase characteristics with the short-time Quaternion Quadratic Phase Fourier Transform (ST-QQPFT) to remove complicated heart dynamics.
- Reduces feature dimensionality using the Success-Based Optimization Algorithm (SBOA) to maximize classification efficiency and generalization performance.
- Learns heart diseases by a Hybrid Structural Graph Attention Network with Spherical Convolutional Neural Network (HS-GAT-SCNN), which is further optimized by the Black-Winged Kite Algorithm (BWKA) to achieve a better predictive quality.
- Obtains diagnostic results with the Adaptive Hash Algorithm with Weighted Probability Model (AHA-WPM) and provides non-recursive storage with a Fair Consensus Blockchain with Heterogeneous Miners (FCB-HM), which finalizes a trusted end-to-end monitoring loop.

## Literature survey

Previous studies focused on securely storing IoMT medical data in the cloud while maintaining data integrity and supporting reliable heart disease analysis are summarized below,

In 2025, Devarajan et al.^16^ presented a more advanced Internet of Medical Things (IoMT) and blockchain-based heart disease monitoring system. Patient and doctor registration were used to initiate system access, followed by key generation and authentication. Physiological signals were measured using IoMT devices and posted to the Interplanetary File System (IPFS). It used a Bloom-Signature Tree Hashing Algorithm (BS-THA) to compute secure hash codes that were stored on the blockchain, and a Message Authentication Code (MAC) was used to verify authentication. The signal conditioning, spectral analysis, Pseudo-Variational Empirical Mode Decomposition (PV-EMD), scalogram, and grayscale conversion, electrocardiogram (ECG) and phonocardiogram (PCG) component extraction, arrhythmia consequence inclusion, and feature optimization were performed, followed by classification with an Optimal Architecture Convolutional Neural Network (OA-CNN). The system effectively and reliably monitored heart disease.

In 2024, Ameen et al.^17^ proposed a cybersecurity system based on blockchain technology to classify the electrocardiogram (ECG) signal in a secure IoMT system. The IoMT devices transmitted cardiac signals, divided into training and test sets. The Pan-Tompkins algorithm was used to extract signal features, and an optimisation algorithm based on a Genetic Algorithm (GA) was used to optimise the features. Testing samples were encrypted using blockchain technology, which enhances confidentiality and tamper resistance during transmission. A Support Vector Machine (SVM) was used for secure verification before classification. It has focused on a workflow that prioritizes secure communication, integrity maintenance, and the reliability of classification in the context of the IoMT. The experimental assessment revealed that clinical repositories at scale increased the system’s strength and diagnostic accuracy.

In 2024, Baseer et al.^18^ proposed an IoMT-based heart disease forecasting workflow that combines physiological sensing and high-order analytical processing. Medical sensors continually recorded the cardiac and hemodynamic indicators via a linked IoMT infrastructure. Structured features were used to address tabular signal representations with TabNet and model the categorical and nonlinear relationships with CatBoost. Predictive modeling incorporated feature refinement and interpretability to reinforce clinical insight. The system architecture facilitated real-time data consumption and adaptive inference on a variety of patient profiles. The unified workflow enhanced predictive accuracy and individualized cardiovascular risk estimation across interconnected healthcare systems.

In 2023, Khanna et al.^19^ recommended a biomedical electrocardiogram (ECG) signal-based Internet of Things (IoT)-based healthcare diagnostic framework. Bidirectional Long Short-Term Memory (BiLSTM) encoding was applied to the features extracted from continuous ECGs recorded by IoT sensors and sent by them. To enhance learning features, Artificial Flora Optimization (AFO) was used to optimize the parameters. Classification was performed using a Fuzzy Deep Neural Network (FDNN), which assigns diagnostic labels based on the extracted signal patterns. Validation of the performance of various feature sets confirmed increased diagnostic capability and greater stability in cardiovascular disease identification.

In 2022, Manimurugan et al.^20^ presented a two-step IoMT-based prediction system for heart disease that combines sensor and imaging data. First, the data collected from physiological sensors were classified using Hybrid Linear Discriminant Analysis with Modified Ant Lion Optimization (HLDA-MALO). If the level of abnormality confidence was low, the echocardiogram images were reassessed using a hybrid Faster Region-based Convolutional Neural Network (Faster R-CNN) with SE-ResNeXt-101. Decision fusion of authenticated results at both phases enhances diagnostic accuracy. The hierarchical flow of work enabled effective resource use and accurate cardiovascular evaluation.

In 2024, Pachiyannan et al.^21^ proposed an electrocardiogram (ECG)- signal-processing-based machine-learning methodology to detect congenital heart disease (CHD). Predictive representations were based on the combination of clinical and demographic characteristics with features derived from ECG analysis. The classification systems examined the structured features’ correlations to differentiate CHD conditions in specific situations, specifically in prenatal healthcare settings. Sensitivity, specificity, accuracy, and receiver operating characteristic curves were used to evaluate diagnostic reliability. The workflow methodology applied focused on the early-detection properties and clinical applicability of maternal and fetal cardiac assessment.

In 2025, Mariam et al.^22^ proposed a personalized cardiac monitoring non-wearable Internet of Medical Things (IoMT) platform. Fixed biomedical sensors received heart rate, oxygen saturation, and electrocardiogram (ECG) signals, and local processing was performed using edge-enabled microcontroller units. Physiological properties were tested using ensemble predictive models trained on clinical repositories. Role-based access was secured, clinician-patient interaction was provided, and an interactive guidance module with personalized recommendations was provided. The architecture enabled heart-rate scaling and real-time diagnostics without the use of wearable technologies. Table 1 provides a comparative summary of existing techniques, highlighting their advantages and limitations in heart disease classification.

**Table 1:** Summary of the existing approaches.

References	Methods	Advantages	Disadvantages
^16^	OA‐CNN	Secure data storage and integrity assurance, high prediction reliability	High computational complexity, increased processing time
^17^	SVM-IoMT	Strong data confidentiality, reliable authentication mechanism	Limited scalability, dependency on handcrafted features
^18^	TabNet-catBoost	Real-time monitoring capability, improved interpretability	High resource consumption, reduced efficiency for large-scale deployment
^19^	IoTDL‐HDD	Effective feature learning improves diagnostic accuracy	Vulnerable to noise variations and a lack of data security mechanisms
^20^	HLDA-MALO	Enhanced diagnostic confidence, multimodal assessment	Increased system complexity, higher computational overhead
^21^	ML-CHDPM	Early disease detection, reduced false diagnosis	Limited consideration of real-time IoMT integration
^22^	XGBoost	Real-time processing, reduced dependence on wearable devices	Limited long-term monitoring flexibility, moderate prediction accuracy

Table 1 provides an overview of current IoMT and blockchain-based methods for monitoring heart disease, which have demonstrated significant advances in secure data acquisition, data integrity, and diagnostic reliability. The adoption of blockchain improved trust and security against tampering, and IoMT enabled continuous physiological tracking. Nevertheless, most of the techniques were characterized by high computational complexity, lacked scalability, were inadequate for handling complex cardiac activity, and underutilized multimodal signals. These restrictions impacted real-time performance, adaptability, and the overall evaluation of heart disease, showing the need for more efficient, scalable, and intelligent monitoring frameworks. Overall, existing techniques for heart disease classification include machine learning models, deep learning architectures, and hybrid approaches integrated with IoMT and blockchain technologies. While machine learning models offer simplicity and interpretability, deep learning methods provide improved feature learning capabilities. Hybrid approaches further enhance performance by combining multiple techniques; however, they often suffer from increased computational complexity and limited scalability. These observations highlight the need for an efficient, scalable, and secure framework, motivating the proposed approach.

### Problem statement

IoMT-based heart disease monitoring systems produce continuous, sensitive physiological data that demand secure storage, reliable transmission, and accurate interpretation. Current techniques often have disadvantages, including incomplete system design, limited incorporation of multimodal heart signals, and limited ability to detect more complex heart rhythm irregularities. Centralized storage systems are susceptible to security attacks, whereas traditional analytical frameworks struggle to handle high-dimensional, noisy biomedical data. The introduction of blockchain increases data security at the expense of latency and computational overhead. Thus, an innovative, streamlined system is needed to provide secure IoMT data management, efficient blockchain interaction, and precise heart disease prediction using innovative deep learning methods, which inspired this paper.

## Proposed methodology

The given HBW-SSG-CNN approach introduces a comprehensive IoMT and blockchain-based system for heart disease observation that is accurate and secure. The cardiac signals are obtained using wearable sensors that feature IoMT and are enhanced with Quasi-Cross Bilateral Filtering (QCBF) to eliminate noise while preserving morphological features. Spectral Envelope-Based Adaptive Empirical Fourier Decomposition (SE-AEFD) is used to decompose nonstationary cardiac components, i.e., the process of signal decomposition. The Short-Time Quaternion Quadratic Phase Fourier Transform (ST-QQPFT) is used to derive discriminative time–frequency and phase features, and these features are optimized using a Success-Based Optimization Algorithm (SBOA) to minimize redundancy and enhance efficiency. The classification of heart diseases is performed using a Hybrid Structural Graph Attention Network with Spherical Convolutional Neural Network (HS-GAT-SCNN), which is then optimized by the Black-Winged Kite Algorithm (BWKA). An Adaptive Hash Algorithm with Weighted Probability Model (AHA-WPM) is used to secure diagnostic outcomes, and the results are stored in a Fair Consensus Blockchain (FCB-HM) to enhances integrity and trust. The proposed methodology will enable precise detection of heart disease using the developed signal processing and deep learning approaches. Adaptive hashing and blockchain consensus mechanisms enhances secure data integrity and decentralized trust.

### IoMT-based cardiac data acquisition

The step of cardiac data acquisition using IoMT triggers the given heart disease monitoring system, allowing it to continuously and in real time gather physiological cardiac signals. At this phase, wearable sensing devices incorporating IoMT are placed on patients to record electrocardiogram (ECG) and phonocardiogram (PCG), which capture the electrical activity and acoustic behavior of the heart, respectively. The sensors work in both resting and ambulatory conditions, allowing cardiac function to be fully monitored in real-life circumstances. The acquired signals are nonstationary and can be affected by motion artifacts, muscle noise, and environmental noise due to patient and sensor motion. After detection, ECG and PCG signals are converted to digital format and sent via secure, low-power wireless protocols to an IoMT gateway or edge processing unit. The communication mechanism has enhanced low latency, low energy consumption, and continuous monitoring. This stage is primarily aimed at enabling non-invasive, remote, continuous observation of heart activity without compromising signal completeness. This stage outputs raw ECG and PCG signal streams, which retain all information about the heart, and sends them to the preprocessing stage to suppress noise and improve the signals.

### Preprocessing using quasi-cross bilateral filtering

In the HBW-SSG-CNN model, Quasi-Cross Bilateral Filtering (QCBF)^23^ is used to preprocess the cardiac signal, enhancing the reliability of cardiac signal analysis before moving on to the decomposition and learning phases.

The signals obtained as raw electrocardiogram and phonocardiogram data from IoMT sensing devices are always subject to baseline drift, motion artifacts, sensor noise, and high-frequency disturbances. These noisy cardiac signals are presented to the QCBF preprocessing stage, where selective smoothing is applied to improve signal quality without loss of diagnostically valuable waveform characteristics. The QCBF mechanism effectively considers both the temporal proximity and similarity of signal amplitudes, enabling the suppression of noise without impairing important cardiac pathways, including ventricular depolarization peaks, repolarization points, and heart-sound boundaries. In contrast to traditional smoothing methods, QCBF actively controls the filtering intensity based on local signal changes, ensuring that sharp edges and clinically significant delimiters are preserved.

The filtered cardiac signal value at a temporal location is computed using the following quasi-cross bilateral formulation based on Eq. (1)

1 $$R_{t} = {\raise0.7ex\hbox{$1$} \!\mathord{\left/ {\vphantom {1 {N_{f} }}}\right.\kern-0pt} \!\lower0.7ex\hbox{${N_{f} }$}}\sum\limits_{{r,s \in W_{t} }} {K_{p} } \left( {\left| {t - r} \right|} \right)K_{q} \left( {\left| {Z_{t} - Z_{r} } \right|} \right)Z_{r}$$

where $R_{t}$ denotes the estimated filtered signal amplitude at time index *t*, $Z_{t}$ represents the original cardiac signal amplitude at the same index, $W_{t}$ defines the temporal neighborhood window centered at *t*, and $N_{f}$​ is a normalization coefficient that enhances amplitude consistency after filtering. The function $K_{p} \left( {\left| {t - r} \right|} \right)$ models the temporal proximity influence and is expressed as Eq. (2)

2 $$K_{p} \left( {\left| {t - r} \right|} \right) = \exp \left( { - {\raise0.7ex\hbox{${\left( {t - r} \right)^{2} }$} \!\mathord{\left/ {\vphantom {{\left( {t - r} \right)^{2} } {C_{p} }}}\right.\kern-0pt} \!\lower0.7ex\hbox{${C_{p} }$}}} \right)$$

where $C_{p}$ controls the width of the temporal smoothing window and determines how neighboring samples influence the central signal value. The amplitude similarity function $K_{p} \left( {\left| {t - r} \right|} \right)$ governs the range filtering behavior and is defined as Eq. (3)

3 $$K_{q} \left( {\left| {Z_{t} - Z_{r} } \right|} \right) = \exp \left( { - {\raise0.7ex\hbox{${\left( {Z_{t} - Z_{r} } \right)^{2} }$} \!\mathord{\left/ {\vphantom {{\left( {Z_{t} - Z_{r} } \right)^{2} } {C_{q} }}}\right.\kern-0pt} \!\lower0.7ex\hbox{${C_{q} }$}}} \right)$$

where $C_{q}$ regulates sensitivity to amplitude variations, enabling suppression of noise while preserving sharp cardiac waveform transitions. These components, when combined, ensures that only samples that are closely aligned in time and have a similar signal magnitude make a significant contribution to the filtered output.

Because of the QCBF operation, low-frequency baseline drift, impulse disturbances, and high-frequency interference can be effectively eliminated. At the same time, the essential morphological and acoustic characteristics of the cardiac signal remain intact. The result of this step is a denoised and baseline-corrected stream of ECG and PCG signals, which is later sent to the spectral-envelope-based adaptive empirical Fourier decomposition stage for frequency-wise analysis.

### Signal decomposition using spectral envelope-based adaptive empirical Fourier decomposition (SE-AEFD)

In HBW-SSG-CNN, Spectral Envelope-Based Adaptive Empirical Fourier Decomposition (SE-AEFD)^24^ is used to decompose pre-processed cardiac signals and analyse their underlying frequency properties.

The signal to this stage is the denoised electrocardiogram and phonocardiogram signals following quasi-cross bilateral filtering. The physiological variability and pathological conditions in these cardiac signals lead to nonlinear and nonstationary behavior, limiting the utility of traditional fixed-basis transforms. SE-AEFD will address this weakness by decomposing the cardiac signal into a finite number of physically relevant frequency-localised components informed by the signal’s spectral envelope. The decomposition process is carried out by converting the filtered cardiac signal into the frequency domain via the discrete Fourier transform, as in Eq. (4)

4 $$U_{c} = \sum\limits_{d = 0}^{D - 1} {V_{d} } \exp \left( { - {\raise0.7ex\hbox{${2\pi rcd}$} \!\mathord{\left/ {\vphantom {{2\pi rcd} D}}\right.\kern-0pt} \!\lower0.7ex\hbox{$D$}}} \right)$$

where $U_{c}$ denotes the complex spectral coefficient at index *c*, $V_{d}$ represents the filtered cardiac signal sample at index *d*, *D* denotes the total signal length, and *r* is a constant frequency scaling factor.

The magnitude spectrum derived from $U_{c}$ it is then analyzed to construct a spectral envelope that adaptively identifies dominant frequency segments. Based on the detected envelope boundaries, the frequency axis is partitioned into multiple non-overlapping subbands, denoted as Eq. (5)

5 $$\Theta_{q} = \left[ {H_{q} ,J_{q} } \right],\,\,\,q = 1,2,.....Q$$

where $\Theta_{q}$ represents the q-th adaptive frequency interval, $H_{q}$ and $J_{q}$ indicates its lower and upper frequency limits, respectively, and *Q* denotes the total number of spectral segments. Each frequency interval corresponds to a specific physiological behavior of cardiac activity, including rhythmic patterns, morphological structures, or pathological oscillations. For each identified interval, an inverse Fourier reconstruction is performed to obtain a frequency-localized component, given by Eq. (6)

6 $$W_{q} \left( t \right) = \sum\limits_{{c \in \Theta_{q} }}^{D - 1} {U_{c} } \exp \left( {{\raise0.7ex\hbox{${2\pi rct}$} \!\mathord{\left/ {\vphantom {{2\pi rct} D}}\right.\kern-0pt} \!\lower0.7ex\hbox{$D$}}} \right)$$

where $W_{q} \left( t \right)$ denotes the q-th decomposed empirical Fourier component in the time domain and ttt represents the temporal index. The original cardiac signal can thus be represented as the sum of all reconstructed components, expressed as Eq. (7)

7 $$X_{t} = \sum\limits_{q = 1}^{Q} {W_{q} } \left( t \right)$$

where $X_{t}$ denotes the reconstructed cardiac signal at time index t. SE-AEFD prevents mode mixing and provides well-separated low-frequency cardiac rhythms, mid-frequency morphological features, and high-frequency pathological variations through adaptive spectral envelope guidance.

Consequently, the product of this step is a collection of empirically decomposed cardiac signal components, all of which bear different spectral and physiological information. These decomposed elements retain temporal locality, frequency adaptiveness, and physical interpretability and are therefore appropriate to further analysis. The resulting signal components are further sent to the feature extraction phase, where time–frequency-phase characterization is performed in detail.

### Feature extraction using short-time quaternion quadratic phase Fourier transform (ST-QQPFT)

In the HBW-SSG-CNN, the Short-Time Quaternion Quadratic Phase Fourier Transform (ST-QQPFT)^25^ is used to extract discriminative representations from decomposed cardiac signal components.

The form of input to this stage is various empirically decomposed components of cardiac signals, obtained at the spectral envelope-based adaptive empirical Fourier decomposition stage. These elements exhibit time-varying frequency and nonlinear phase properties, so localized time–frequency-phase analysis is required. To meet this need, ST-QQPFT divides each decomposed signal into short-time frames and transforms them into a quaternion-valued format that allows the simultaneous description of amplitude changes, instantaneous frequency changes, phase modulation, and inter-component dependencies. Quadratic phase modelling also increases sensitivity to transient cardiac events and sudden morphological changes that linear spectral transforms tend to miss.

The short-time quaternion quadratic phase representation of a decomposed cardiac component is expressed as Eq. (8)

8 $$Y_{u} \left( {v,t} \right) = \int {R_{c} } \left( \tau \right)M\left( {t - \tau } \right)\exp \left( { - P_{1} \tau^{2} - P_{2} \tau v} \right)d\tau$$

where $Y_{u} \left( {v,t} \right)$ denotes the quaternion-domain transform coefficient at time index *t* and frequency index *v*, $R_{c} \left( \tau \right)$ represents the input decomposed cardiac signal sample at temporal index *τ*, $M\left( {t - \tau } \right)$ is a localized window function controlling temporal resolution, and $P_{1}$ and $P_{2}$ are quadratic phase control constants governing nonlinear phase sensitivity and frequency modulation strength, respectively.

To form a compact and discriminative feature representation suitable for learning, statistical descriptors are derived from the magnitude of the quaternion coefficients across time–frequency planes, expressed as Eq. (9)

9 $$F_{d} = \sum\limits_{t = 1}^{T} {\sum\limits_{v = 1}^{V} {\left| {Y_{u} \left( {v,t} \right)} \right|^{2} } }$$

where $F_{d}$ denotes the extracted feature energy for a given decomposed component, *T* and *V* represent the total number of time frames and frequency bins, respectively. This model represents local time-varying and global spectral distributions of cardiac activity and, additionally, is resistant to noise and signal variability.

Consequently, the ST-QQPFT generates a high-dimensional feature set of vectors that captures both detailed time–frequency phase properties of ECG and PCG signals in a single mathematical expression. These extracted features offer high discriminatory capacity for analysing cardiac conditions. The resulting feature vectors are then sent to the Success-Based Optimization Algorithm for feature selection to remove redundancy and improve classification efficiency.

### Feature selection using success-based optimization algorithm (SBOA)

In the HBW-SSG-CNN model, the Success-Based Optimization Algorithm (SBOA) is used to select high-dimensional cardiac feature representations into a small, informative subset^26^.

The input to this stage is feature vectors, which are the result of a short-time quaternion quadratic-phase Fourier transform, and each feature vector is a localized and global cardiac feature. As time–frequency-phase representations are large-dimensional and redundant, direct classification can increase computational cost and decrease generalization. SBOA is a method that can be used to overcome this limitation, with candidate feature subsets evaluated to assess their contribution to classification success and iteratively enforced on those that improve predictive performance. To initialize the population of candidate solutions, the algorithm first forms each solution as an encoded feature selection mask, which is a binary number as shown in Eq. (10)

10 $$G_{u} = \left[ {J_{u,1,} J_{u,2,...,} J_{u,R} } \right]$$

where $G_{u}$ denotes the u-th candidate solution, $J_{u,R}$ represents the selection state of the *R*-th feature, and RRR is the total number of extracted features.

The effectiveness of each candidate solution is evaluated using a success-driven fitness function that balances classification accuracy and feature compactness, defined as Eq. (11)

11 $$E_{u} = \Omega_{1} \cdot Q_{u} + \Omega_{2} \cdot \left( {1 - {\raise0.7ex\hbox{${S_{u} }$} \!\mathord{\left/ {\vphantom {{S_{u} } R}}\right.\kern-0pt} \!\lower0.7ex\hbox{$R$}}} \right)$$

where $E_{u}$ denotes the fitness score of the candidate solution $G_{u}$, $Q_{u}$ represents the classification success rate achieved using the selected feature subset, $S_{u}$ is the number of selected features, and $\Omega_{1}$ and $\Omega_{2}$ are weighting constants controlling the influence of performance and dimensionality reduction. The candidate solutions with higher fitness values are deemed more successful and influence subsequent changes.

Consequently, the SBOA generates a subset of features that are minimally redundant and provide the best cardiac information while removing redundancy and noise. The chosen characteristics minimize computational load and alleviate overfitting in later learning steps. The reduced versions of the feature vectors found after this step are then passed to the deep learning-trained classification model to be able to predict heart disease.

### Hybrid Black-winged spherical structural graph convolution neural network (HBW-SSG-CNN) for classification

After feature selection, the selected cardiac features are then fed into a Hybrid Structural Graph Attention Network^27^ using a Spherical Convolutional Neural Network^28^ (HS-GAT-SCNN) to classify heart disease. The input to this step is a set of discriminative, reduced-dimensional feature vectors obtained via a success-based optimization algorithm, with each feature vector containing localized and global cardiac features derived from ECG and PCG signals. To successfully capture inter-feature dependencies and structural relationships in cardiac dynamics, the specified features are initially placed in a graph system, where nodes correspond to the particular cardiac features, and the relationships between them are modeled as edges representing temporal and physiological correlations. In simple terms, the HS-GAT-SCNN model learns relationships between cardiac features using graph-based attention while capturing spatial patterns through spherical convolution, enabling more accurate disease classification.

#### Structural graph attention network

To simulate significant structural interdependencies among cardiac characteristics within a single IoMT blockchain healthcare setting, the HBW-SSG-CNN framework is trained using Hybrid Structural Graph Attention Network classification, such that interactions between features are learnt in a graph-based, attention-controlled manner to increase diagnostic accuracy.

Suppose the optimised feature set of the last Success-Based Optimization Algorithm is a graph whose nodes are identified by the selected cardiac feature, and whose relationships are represented by statistical or physiological values. The starting node representation of the matrix is given by Eq. (12).

12 $$R_{uv}^{\left( 1 \right)} = relu\left( {d_{u}^{\left( 1 \right)} \cdot T_{{}}^{\left( 1 \right)} \cdot d_{v}^{\left( 1 \right)} } \right)$$

where $T_{{}}^{\left( 1 \right)}$ is a learnable transformation matrix controlling structural interaction strength, and relu denotes the rectified linear activation applied to preserve positive relevance. This formulation enables the network to suppress weak or redundant dependencies while amplifying diagnostically significant feature relations.

The normalized attention weight assigned to each neighbouring node is then expressed as Eq. (13)

13 $$W_{uv}^{\left( 1 \right)} = \frac{{\exp \left( {R_{uv}^{\left( 1 \right)} } \right)}}{{\sum\nolimits_{v \in N\left( u \right)} {\exp \left( {R_{uv}^{\left( 1 \right)} } \right)} }}$$

where $N\left( u \right)$ represents the neighbourhood set of a node *u*. This normalization enhances stability and comparability of attention scores across different node degrees within the graph.

Using the learned attention weights, the updated node representation is obtained through weighted aggregation as in Eq. (14)

14 $$d_{u}^{\left( 2 \right)} = \sum\nolimits_{v \in N\left( u \right)} {W_{uv}^{\left( 1 \right)} \cdot d_{u}^{\left( 1 \right)} }$$

where $d_{u}^{\left( 2 \right)}$ captures both local structural context and global relational importance, yielding a refined feature embedding that better reflects cardiac interdependencies.

To guide the learning process toward optimal classification performance, a structural fitness objective is defined as Eq. (15).

15 $$F_{{}}^{\left( 1 \right)} = {\raise0.7ex\hbox{$1$} \!\mathord{\left/ {\vphantom {1 U}}\right.\kern-0pt} \!\lower0.7ex\hbox{$U$}}\sum\limits_{u = 1}^{U} {\left\| {d_{u}^{\left( 2 \right)} - d_{u}^{\left( 1 \right)} } \right\|^{2} }$$

The fitness value describes how consistent and stable attention-driven feature refinement is, so that structural enhancement does not introduce distortion, while ensuring better discriminative capacity.

The HSGAN generates context-sensitive, interpretable graph-level representations through a hybrid attention-based structural learning process that effectively encodes relationships among diagnostically important features of cardiac data. The resulting refined embeddings are then passed to the Spherical Convolutional Neural Network phase of deep spatiotemporal pattern learning and eventual heart disease identification.

#### Spherical convolutional neural network (SCNN)

The current step proposes implementing a spherical convolutional neural network (SCNN) as part of HBW-SSG-CNN to acquire rotation-aware representations of structured cardiac descriptors in a spherical space^29^. The SCNN acts on the output of the hybrid graph attention module, which uses spherical feature maps to enable consistent analysis of spatial structural patterns regardless of orientation.

Let the spherical input signal be denoted as $U_{{}}^{\left( 1 \right)}$, where each point corresponds to cardiac feature responses on a unit sphere. The spherical convolution operation is expressed as Eq. (16)

16 $$R^{1} U_{{}}^{\left( 1 \right)} * V^{1}$$

where $V^{1}$ represents the learnable spherical kernel and * denotes spherical correlation. This operation preserves rotational consistency by aligning kernel responses across all orientations.

To enhances equivariant feature transformation, the rotated response satisfies Eq. (17)

17 $$R_{rot}^{1} = Q^{1} \left( H \right)R^{1}$$

where $Q^{1} \left( H \right)$ denotes the rotation operator corresponding to a spatial transformation *H*. Nonlinear activation is then applied to enhance discriminative capacity as in Eq. (18)

18 $$R^{2} = \varphi \left( {R^{1} } \right)$$

with $\varphi \left( \cdot \right)$ indicating a bounded activation function that maintains stability across layers.

Finally, the invariant descriptor is obtained through spherical pooling, defined as Eq. (19)

19 $$R^{3} = \Omega \left( {R^{2} } \right)$$

where $\Omega \left( \cdot \right)$ aggregates global spherical responses into a compact representation. Here, $R^{3}$ captures rotation-invariant cardiac characteristics essential for robust diagnosis.

**Fig. 1:**
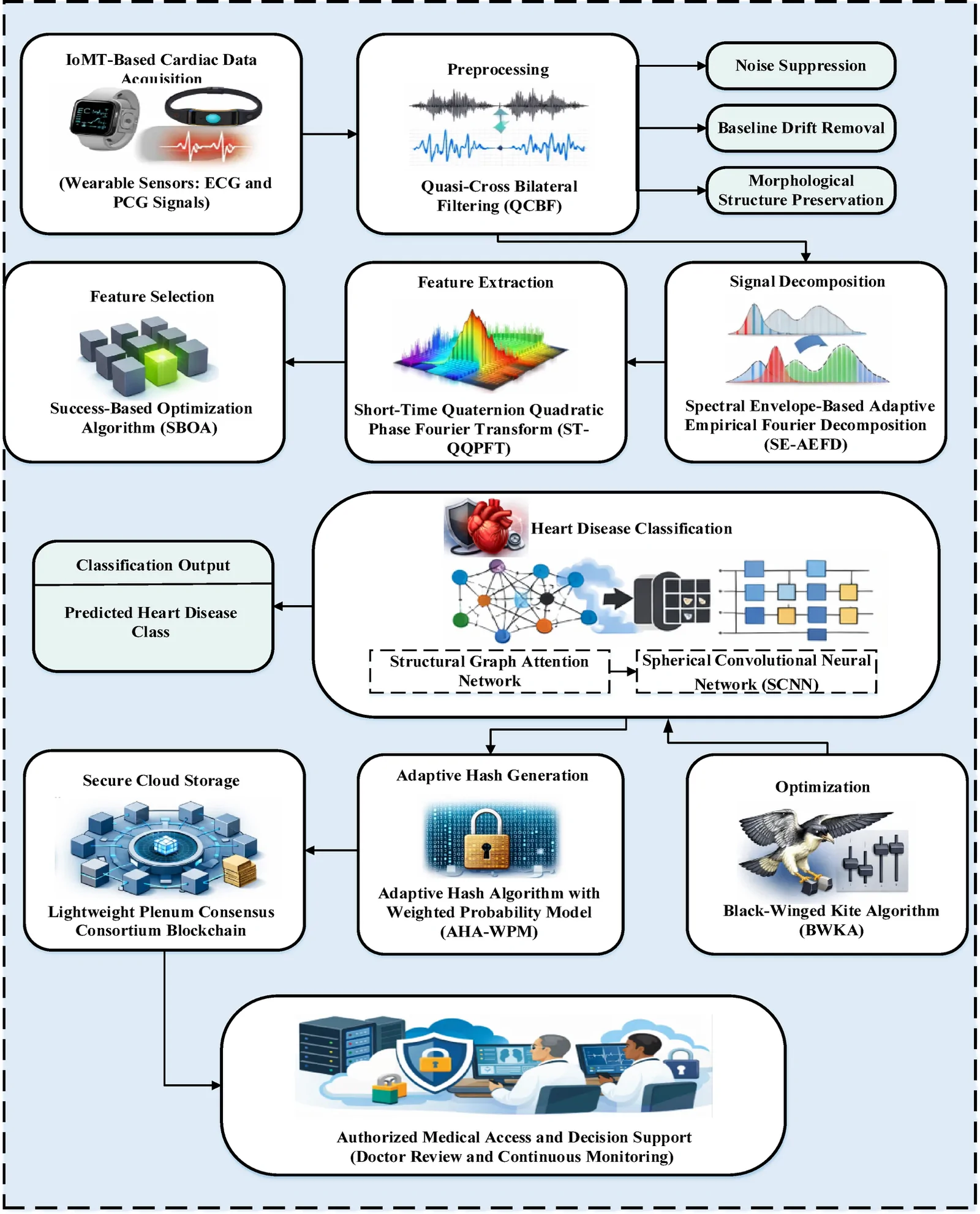
Workflow of proposed HBW-SSG-CNN methodology.

This SCNN phase produces small, orientation-sensitive feature embeddings, which are then passed to the optimization section to optimize their parameters and enhance their performance in the IoMT and Blockchain-Based Heart Disease Monitoring System via Deep Learning. The workflow of the proposed methodology is depicted in Fig. 1.

#### Black-Winged kite algorithm (BWKA)

**Fig. 2:**
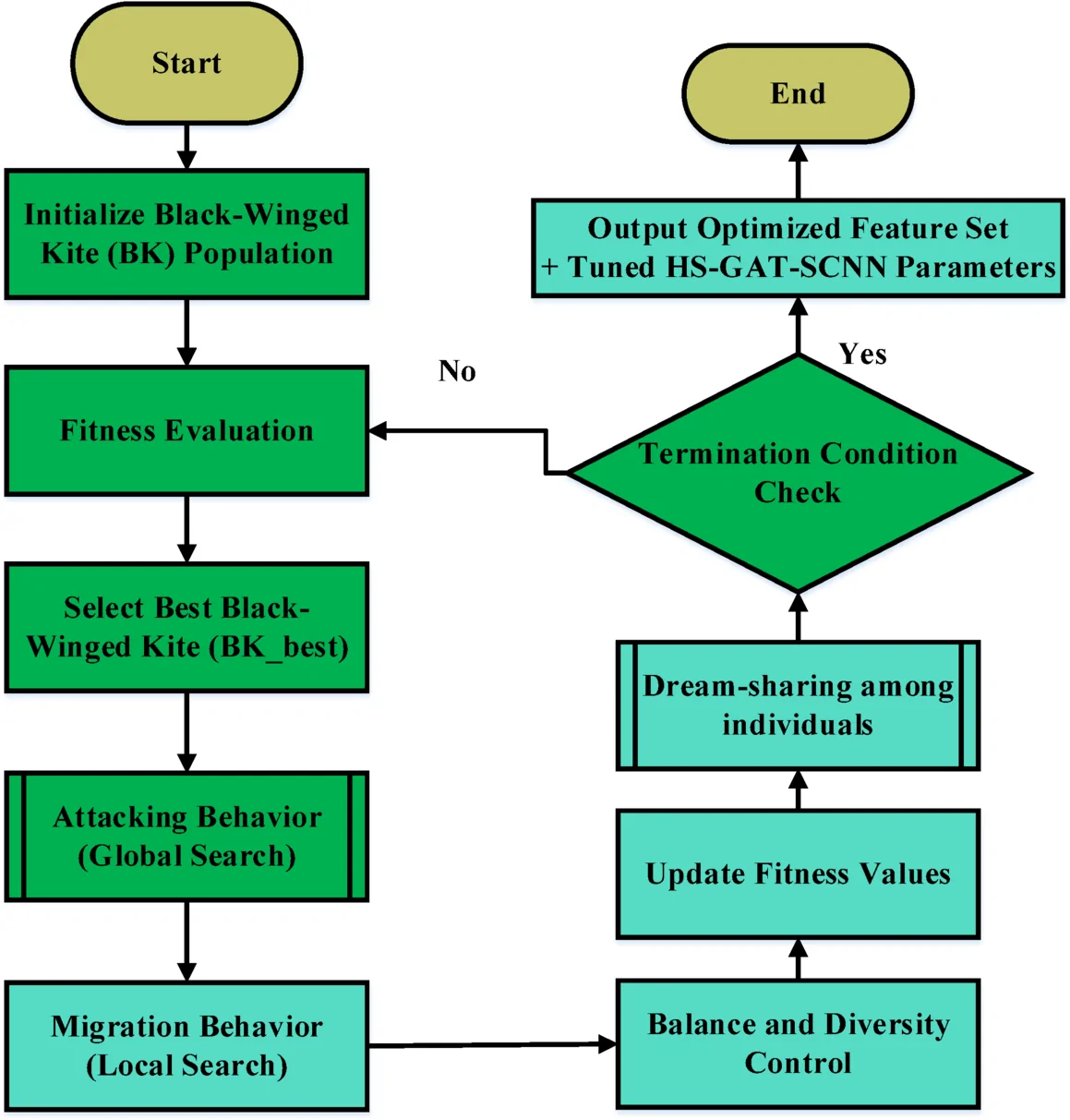
Flowchart for BKWA.

The Black-Winged Kite Algorithm is used as an optimization technique to automatically fine-tune model parameters, improving prediction accuracy and stability. The input to this process is the trained network parameters, along with the validation performance indicators obtained in the previous classification process. BWKA is used to model the adaptive search behavior of black-winged kites, in which candidate solutions are repeatedly updated to balance global exploration and local exploitation, preventing premature convergence and unstable learning. The BKWA flowchart is shown in Fig. 2.

Let the population matrix be represented as Eq. (20)

20 $$B_{1} = \left[ {u_{pq} } \right],\,\,p = 1,2,....,P,\,\,q = 1,2,...Q$$

where *P* denotes the number of candidate solutions and *Q* represents the number of tunable hyperparameters. The position of each candidate is initialized as in Eq. (21)

21 $$u_{pq} = l_{q} + \,\,v_{pq} \left( {h_{q} - l_{q} } \right)$$

where $l_{q}$​ and $h_{q}$ define the lower and upper bounds of the *q* th hyperparameter, and $v_{pq}$ is a uniformly distributed random scalar in the range $\left[ {0,1} \right]$. The quality of each candidate is evaluated using a fitness function defined as Eq. (22)

22 $$u_{p}^{new} = u_{p} + \,\,r_{p} \left( {u_{best} - u_{p} } \right)$$

where $\,r_{p}$ is a control coefficient regulating the search step, and $u_{best}$ is the current optimal solution.

BWKA can optimize hyperparameters to enhance the accuracy, speed, and stability of the classifier by refining the set of hyperparameters used. The optimized model outputs are, in turn, sent to the adaptive hash algorithm, which uses the weighted probability model to integrate secure blockchain.

### Adaptive hash algorithm based on weighted probability model (AHA-WPM)

The adaptive hashing mechanism converts diagnostic outputs into secure hash values, helping to protect medical data from unauthorized modification. The stage ensures that diagnostic outputs are represented safely in the HBW-SSG-CNN framework using an Adaptive Hash Algorithm Based on the Weighted Probability Model (AHA-WPM)^30^. The input to the process is optimized classification decisions and related cardiac records that are generated by the deep learning classifier. To improve dynamic response to data sensitivity and security requirements, AHA-WPM calculates hash values by assigning adaptive weights to data symbols based on their likelihood of occurrence, which is suitable for the context of IoMT.

Let the processed data sequence be denoted as Eq. (23)

23 $$D_{1} = u\left[ {r_{t} } \right],\,\,t = 1,2,..,T$$

where *T* represents the data length and $r_{t}$ denotes the * t*-th data symbol. The weighted probability of each symbol is defined as Eq. (24)

24 $$w_{t} = {\raise0.7ex\hbox{${u_{t} }$} \!\mathord{\left/ {\vphantom {{u_{t} } {\sum\nolimits_{c = 1}^{T} {u_{c} } }}}\right.\kern-0pt} \!\lower0.7ex\hbox{${\sum\nolimits_{c = 1}^{T} {u_{c} } }$}}$$

where $u_{t}$ indicates the assigned importance weight of the * t* th symbol. The adaptive information measure guiding hash length is computed as Eq. (25)

25 $$H_{d} = - \sum\limits_{t = 1}^{T} {w_{t} } \log \left( {w_{t} } \right)$$

where $H_{d}$ reflects the weighted uncertainty of the data. Based on this measure, the adaptive hash length is obtained as Eq. (26)

26 $$L_{d} = \eta \cdot H_{d}$$

where *η* is a scaling factor that controls the security strength. The final hash value is generated as Eq. (27)

27 $$Z_{d} = \psi \left( {D_{1} ,L_{d} } \right)$$

where $\psi \left( \cdot \right)$ denotes the adaptive hashing function.

Characters AHA-WPM creates small but collision-free hash codes that maintain the integrity of data with fewer computational expenses through weighted probability-guided adaptation. The adaptive hash values are then sent to the Fair Consensus Blockchain of Heterogeneous Miners (FCB-HM) to facilitate safe and reliable transactions of medical data.

### Fair consensus blockchain with heterogeneous miners (FCB-HM)

The blockchain consensus mechanism ensures fair and efficient validation of medical data across distributed nodes, improving trust and reliability in data storage. At this phase, secure data persistence will be achieved by incorporating the Fair Consensus Blockchain with Heterogeneous Miners (FCB-HM)^31^ into the HBW-SSG-CNN system. The input to this process is the adaptive hash-coded cardiac records created in the last hashing step and sent to the blockchain for validation and storage. The suggested consensus mechanism evenly distributes mining work across nodes with varying computational power, minimizing confirmation delays and preventing miner monopolies without sacrificing decentralization^32^.

Let the set of participating miners be expressed as Eq. (28)

28 $$M_{1} = \left\{ {d_{u} } \right\},\,u = 1,2,.....U$$

where *U* denotes the total number of heterogeneous miners and $d_{u}$ represents the computational capability of the *u* th miner. The fair participation weight assigned to each miner is defined as Eq. (29)

29 $$w_{u} = \frac{{d_{u} }}{{\sum\nolimits_{c = 1}^{U} {d_{c} } }}$$

which enhances proportional yet balanced influence in block generation. The probability of miner selection for block validation is computed as Eq. (30)

30 $$p_{u} = \frac{{w_{u} }}{\max \left( w \right)}$$

where $\max \left( w \right)$ denotes the maximum miner weight in the network. The block acceptance fitness is evaluated as Eq. (31)

31 $$B_{v} \arg \min \left( {F_{b} } \right)$$

ensuring fairness and efficiency in consensus.

**Fig. 3:**
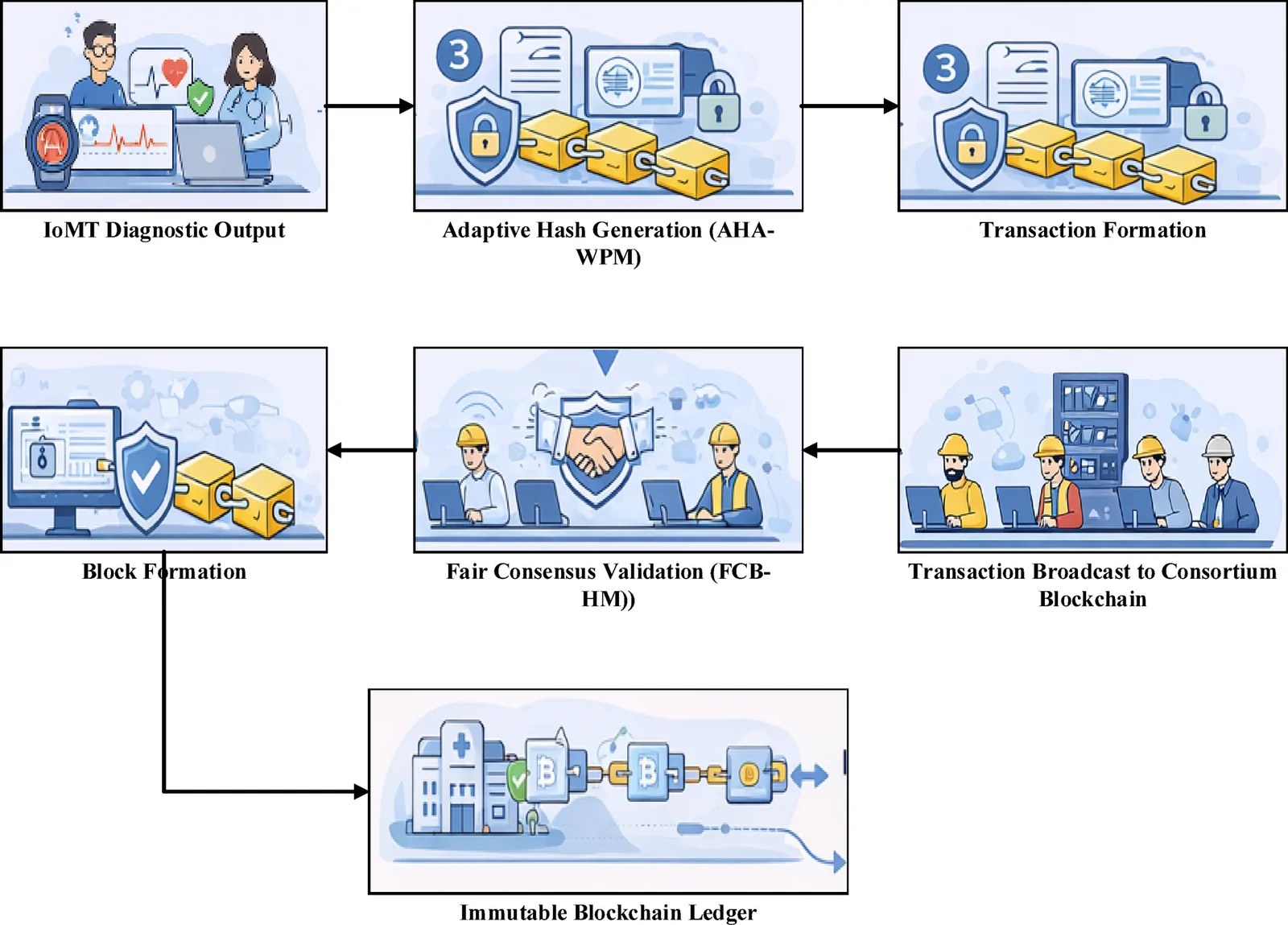
Process of fair consensus blockchain with heterogeneous miners (FCB-HM).

By this process that is shown in Fig. 3, FCB-HM will be able to enhances the unchanged, transparent, and unaltered storage of cardiac monitoring records and facilitate fair participation by miners. The blockchain ledger created during this step is validated and then used in the results section to perform performance evaluation and reporting.

## Result and discussion

This part presents the experimental assessment and comparative study conducted to verify the dependability, strength, and efficiency of the proposed IoMT and Blockchain-Based Heart Disease Monitoring System using HBW-SSG-CNN. The entire architecture is deployed and run on the Python computational platform, which comprises a signal processing module, a deep learning module, an optimization module, and a a blockchain module to evaluate real-time cardiac monitoring performance^33,34^. Table 2 indicates Simulation Parameters.

**Table 2:** Simulation parameters.

Parameter description	Value/setting
Simulation environment	Python
Dataset size	PhysioNet and Cleveland Clinic
Training data ratio	80%
Testing data ratio	20%
Signal types	ECG, PCG
Sampling frequency	360 Hz
Signal duration	10 s
Number of training epochs	100
Batch size	32
Learning rate	0.001
Optimization Iterations	50
Population size	30
Classification type	Multi-class
Evaluation metrics	Accuracy, sensitivity, specificity, F1-score

To enhances robust and reliable evaluation, fivefold stratified cross-validation is incorporated in addition to the conventional 80/20 train-test split. This approach reduces variance in performance estimation and improves generalization, particularly for small-scale medical datasets. The experimental setup is implemented using the Python platform, integrating signal preprocessing, feature extraction, optimization, classification, and blockchain modules. The ECG and PCG signals are sampled at 360 Hz and processed through the proposed pipeline. The dataset is divided into training and testing sets using an 80:20 ratio, and fivefold cross-validation is additionally employed to ensure robust performance evaluation. The model is trained for 100 epochs with a batch size of 32 and a learning rate of 0.001. Performance is evaluated using standard metrics, including accuracy, sensitivity, specificity, and F1-score.

### Dataset description

The proposed structure is tested on two publicly available benchmark datasets: the PhysioNet cardiac signal repository and the Cleveland Clinic heart disease dataset, as mentioned in the reference section. The study utilizes publicly available cardiac signal datasets from PhysioNet, including benchmark electrocardiogram (ECG) and phonocardiogram (PCG) recordings (e.g., PhysioNet/CinC Challenge datasets), consisting of clinically annotated signal records collected under varying physiological and environmental conditions. These signals represent diverse cardiac rhythms and heart sound patterns, making them suitable for evaluating signal-level robustness and feature discrimination. It is important to note that the PhysioNet dataset provides waveform-based ECG and PCG signals, whereas the Cleveland dataset consists of structured clinical attributes. These datasets represent different modalities and are evaluated independently to assess both signal-based and clinical feature-based diagnostic performance. The Cleveland dataset contains 303 samples with multiple clinical attributes related to heart disease diagnosis, while the PhysioNet dataset includes annotated cardiac signal recordings used for evaluating signal processing robustness.

The Cleveland Clinic heart disease data set contains structured clinical attributes related to heart conditions, including the type of chest pain, the resting electrocardiogram, evidence of shortness of breath, evidence of abnormal heart rate, and evidence of arrhythmia. These features aid in classifying multi-class heart diseases and improve the generalizability of the diagnosis.

To enhances consistency across experiments, 80 percent of the total samples will be used for training, and the remaining 20 percent for testing. Such a division will provide sufficient capacity for learning and unbiased performance appraisal. The PhysioNet ECG and PCG signals are pre-processed with the Quasi-Cross Bilateral Filtering, and spectral and decomposition analyses are performed with normalization and baseline correction.

Table 3 summarizes the visual results of the various processing stages: raw signal input, noise suppression, normalization, baseline drift correction, spectral envelope analysis, SE-AEFD-based signal decomposition, and scalogram generation. These image-level findings corroborate sound noise reduction, sharper signals, and a better time–frequency representation, which play direct roles in proper feature extraction and classification in the later stages^35^.

**Table 3:**
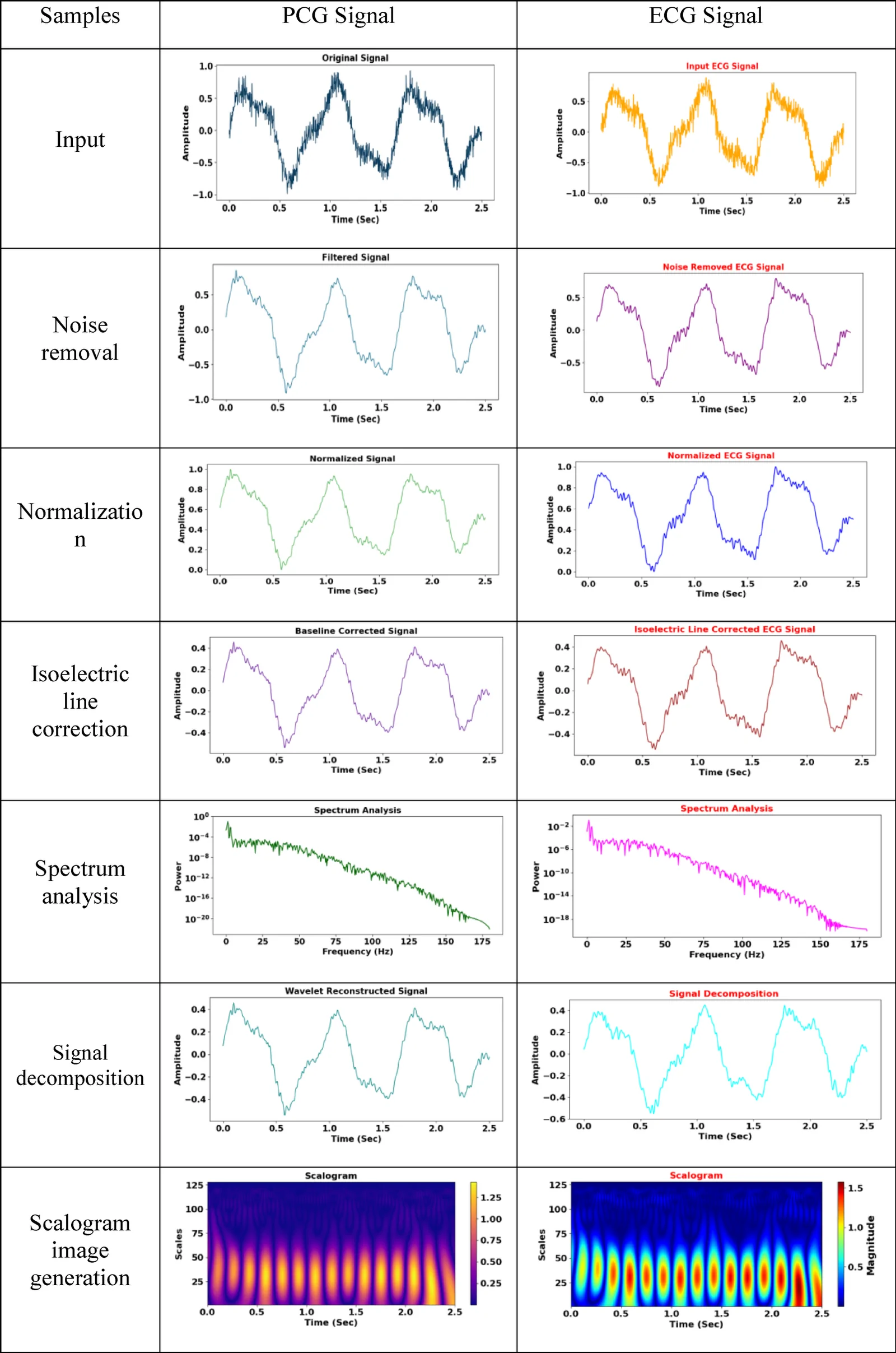
Sample output results obtained at different stages.

By and large, the data variety and preprocessing results provide a solid basis for authenticating the proposed HBW-SSG-CNN model in a real-life setting for IoMT-enabled healthcare. The Cleveland dataset consists of 303 samples with multiple diagnostic classes. The dataset is approximately balanced across classes, and stratified sampling is applied to preserve class distribution during training and testing. The PhysioNet datasets are publicly available at https://physionet.org/, and the Cleveland heart disease dataset can be accessed through the UCI Machine Learning Repository (https://archive.ics.uci.edu/). These datasets are widely used benchmarks in cardiovascular disease research.

### Performance analysis

**Fig. 4:**
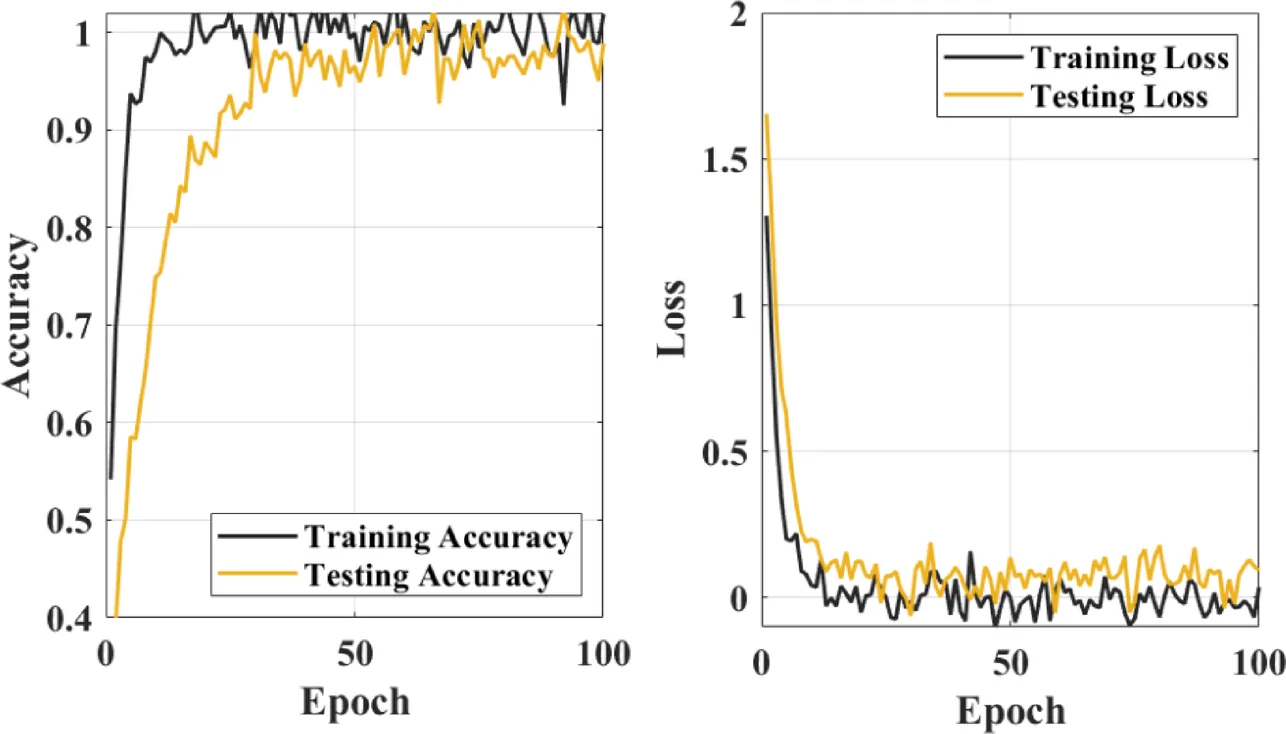
Training and testing accuracy and loss curves of the proposed HBW-SSG-CNN model.

The performance analysis of HBW-SSG-CNN is discussed here:

The convergence behaviour of the proposed HBW-SSG-CNN model is depicted in Fig. 4, which shows training and testing results across different epochs. The precision curves exhibit fast learning in the first epochs, followed by convergent learning, which implies successful learning and good generalization. At the same time, the loss curves decline rapidly, remain low, and exhibit slight swings, indicating efficient error reduction and decreased overfitting. The strong correspondence between the training and test trends is a strong indication of the robustness and reliability of the proposed classification framework^36^.

**Fig. 5:**
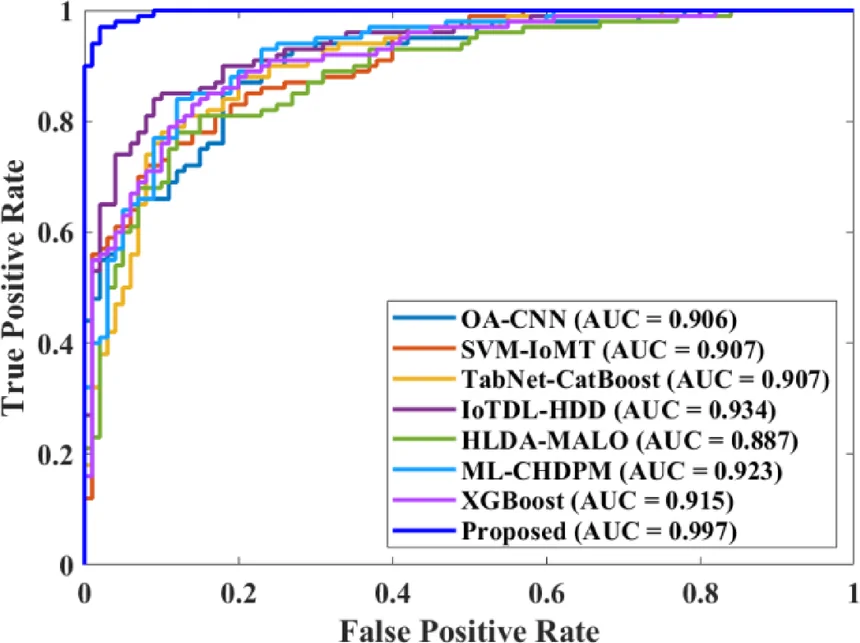
ROC curve comparison of the proposed model with existing methods.

Figure 5 shows the Receiver Operating Characteristic (ROC) curves for the proposed HBW-SSG-CNN model and several existing methods for classifying heart disease. The proposed model has the highest Area Under the Curve (AUC) of 0.997, indicating better discrimination between normal and abnormal cardiac conditions. The curve is always found to be closer to the top-left corner, indicating a high true positive rate and a low false positive rate. This performance demonstrates the strength, stability, and greater diagnostic power of the proposed framework compared to benchmark approaches^37^.

**Fig. 6:**
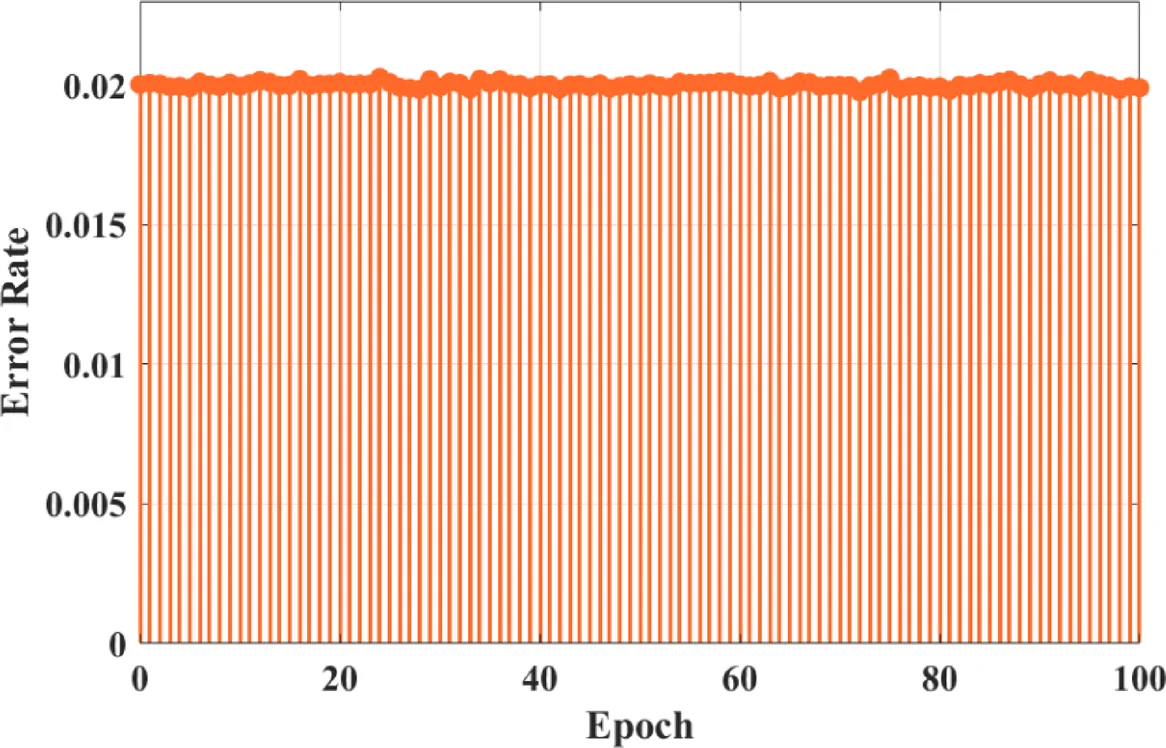
Error rate convergence across training epochs.

Figure 6 shows how the error rate changes with the number of training epochs for the proposed classification model. The error rate is neither elevated nor fluctuating during the training process, indicating successful learning and a high ability to generalize. The lack of strong fluctuations is an indicator of strong resistance to overfitting and training instability. This tendency demonstrates that the optimized deep learning architecture is efficient and consistent in its performance, achieving high accuracy even after a long training period for heart disease classification.

**Fig. 7:**
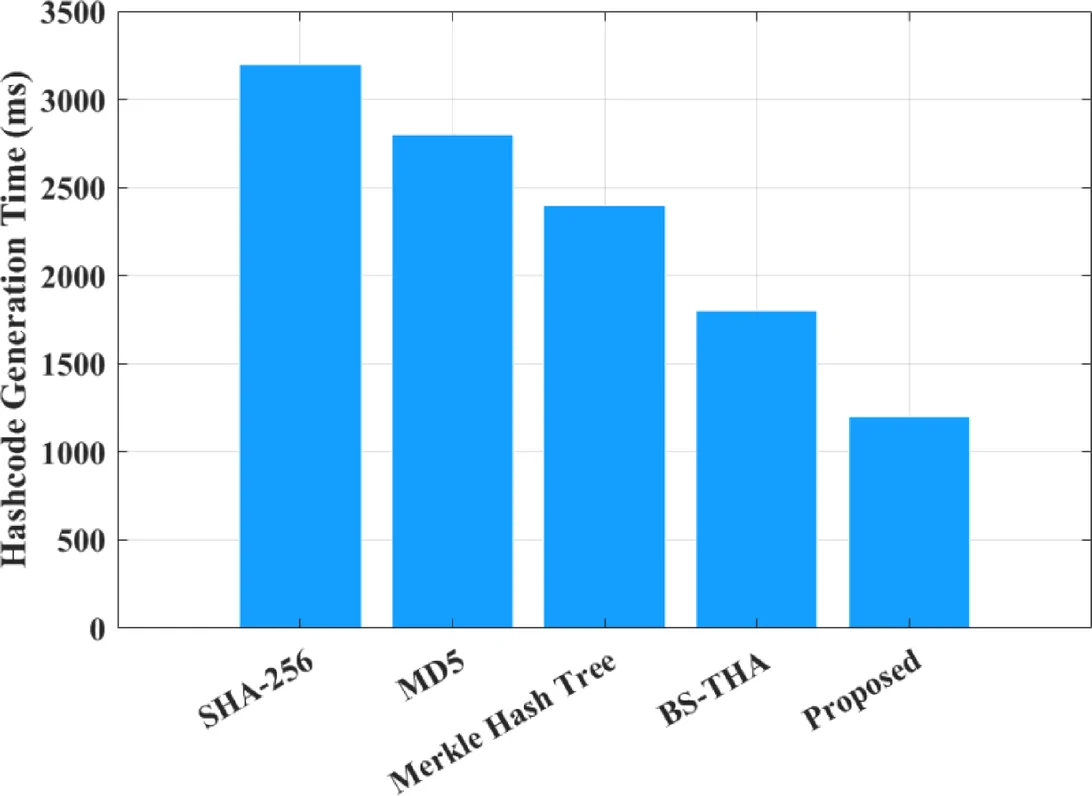
Comparison of hash code generation time for different hashing techniques.

Figure 7 presents the results of a comparative analysis of the time required to generate a hash code using various hashing algorithms, including SHA-256, MD5, Merkle Hash Tree, BS-THA, and the suggested algorithm. The proposed solution has the shortest hash-generation time, demonstrating its computational efficiency and suitability for real-time IoMT-based healthcare scenarios. The shorter latency of hash generation enables faster data authentication and processing of blockchain transactions, which is essential in heart disease monitoring applications where a quick response is required, and data integrity must be safeguarded^38^.

**Fig. 8:**
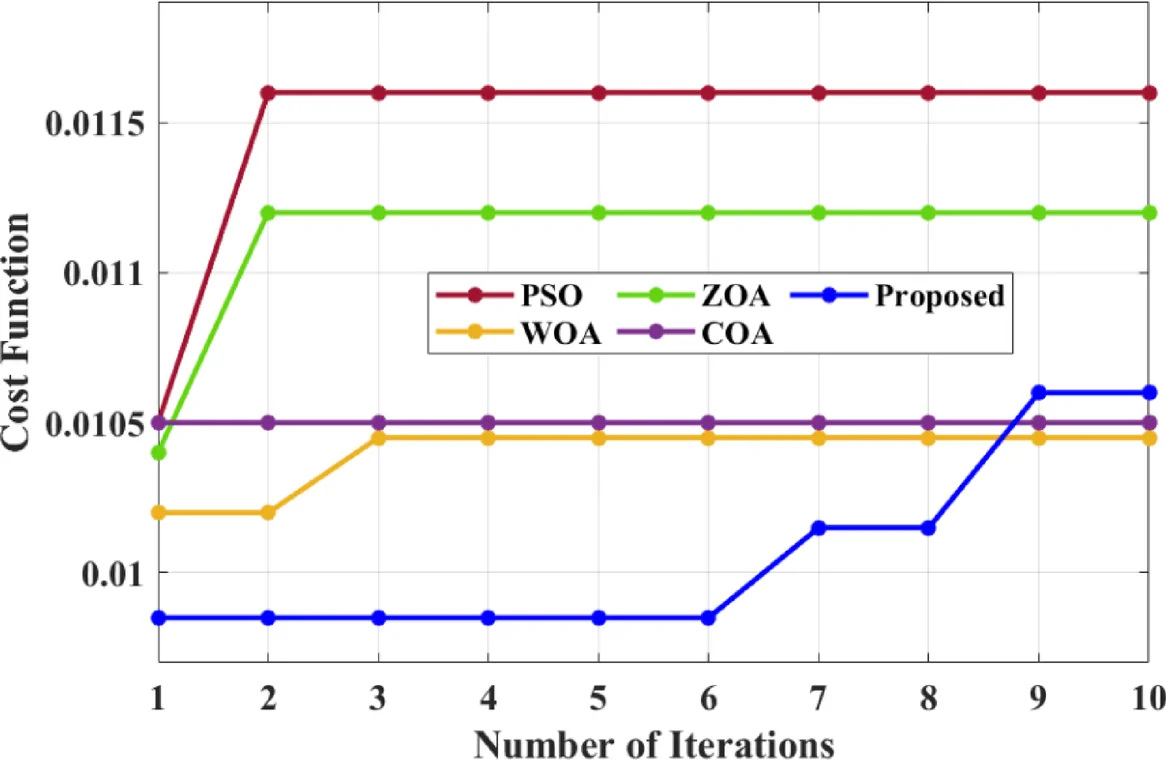
Cost function convergence analysis of optimization algorithms.

The convergence of the cost functions of different algorithms in optimization is shown in Fig. 8 as a function of the number of iterations. The suggested optimization plan always has a lower cost than PSO, WOA, ZOA, and COA. The convergence is quick and steady, suggesting efficient search space exploration and exploitation. This enhanced convergence behaviour is an added advantage towards better model tuning, training stability, and classification accuracy in the proposed heart disease monitoring model.

A comparative analysis of heart disease classification methods based on key performance indicators, such as TPR, TNR, FPR, FNR, and PPV, is presented in Table 4. The HBW-SSG-CNN model achieves the best TPR and TNR, and the lowest FPR and FNR, indicating its strong detection performance and minimal misclassification. The enhanced PPV indicates trustworthy, favorable forecasts. All in all, the findings confirm that the suggested framework is superior in terms of accuracy, robustness, and diagnostic reliability compared to conventional and hybrid frameworks^39^.

**Table 4:** Comparative performance analysis of heart disease classification techniques.

Technique	TPR (%)	TNR (%)	FPR	FNR	PPV (%)
OA-CNN	98.30	98.48	1.5026	1.6032	98.87
SVM-IoMT	96.74	96.20	3.0175	3.2154	96.54
TabNet-CatBoost	95.12	94.30	5.6302	4.2036	94.20
IoTDL-HDD	93.23	91.60	8.0693	6.8753	92.95
HLDA-MALO	90.21	89.62	10.2145	9.1248	90.12
ML-CHDPM	92.85	91.10	7.4521	6.2034	91.80
XGBoost	94.67	93.92	5.9823	5.1046	93.40
HBW-SSG-CNN (Proposed)	99.21	99.08	0.92	0.98	99.02

Table 5 compares conventional and advanced hashing methods in terms of hash generation efficiency. The suggested AHA-WPM has the minimum time for hash generation and computation cost, and the probability of collision is very low. Older hashing algorithms like MD5 are much faster to compute but have poor security, whereas newer algorithms like SHA-256 offer high security at the cost of higher latency. This is because the proposed approach provides a good compromise among speed, security strength, and collision resistance by dynamically varying the hash length based on a weighted probability model; thus, it is well-suited to the context of IoMT-enabled blockchain healthcare systems.

**Table 5:** Comparative analysis of hash generation performance.

Hashing technique	Hash generation time (ms)	Collision rate	Computational cost	Security strength
SHA-256	3200	Medium	High	High
MD5	2800	High	Low	Low
Merkle Hash Tree	2400	Medium	Medium	High
BS-THA	1800	Low	Medium	High
AHA-WPM (Proposed)	1200	Very Low	Low	Very high

Table 6 presents a comparative analysis of blockchain consensus mechanisms based on throughput, latency, energy use, scalability, and security. Traditional solutions like PoW offer high security but suffer from excessive latency and consume too much energy to be helpful in an IoMT setup. The proposed FCB-HM consensus achieves the best transaction throughput and latency while minimizing energy consumption. It enables heterogeneous miners, thus being just, scalable, and highly secure, making it very useful for real-time, secure healthcare data management.

**Table 6:** Comparative analysis of blockchain performance metrics.

Consensus technique	Throughput (TPS)	Transaction latency (ms)	Energy consumption	Scalability	Security level
Proof of Work (PoW)	7	3200	Very high	Low	High
Proof of Stake (PoS)	45	1600	Medium	Medium	High
Delegated PoS (DPoS)	120	980	Low	High	Medium
PBFT	150	850	Medium	Low	High
BS-THA Blockchain	210	620	Low	Medium	High
FCB-HM (Proposed)	320	410	Very Low	Very high	Very high

Table 7 emphasizes the influence of feature selection on classification performance and efficiency. Before feature selection, the high-dimensional feature space increases computational complexity and memory consumption, thereby reducing classification accuracy. Using the Success-Based Optimization Algorithm-based feature selection, it has been observed that the number of features is significantly reduced and accuracy is enhanced. The smaller feature set allows for faster, lower memory use, with no loss of information. These findings indicate that feature selection optimization is vital towards improving the efficiency, stability, and diagnostic accuracy of models^40^ (Table 8).

**Table 7:** Impact of feature selection on model performance.

Feature set	Number of features	Classification accuracy (%)	Computation time (s)	Memory usage (MB)
Before selection	512	95.84	2.86	148
After selection (SBOA)	176	99.21	1.42	79

**Table 8:** Overall performance comparison of heart disease monitoring methods.

Techniques	Accuracy (%)	Sensitivity (%)	Specificity (%)	F1-score (%)	Computation time (s)
SVM-IoMT	92.14	91.30	92.05	91.72	2.94
TabNet-CatBoost	94.87	94.10	94.62	94.36	2.61
IoTDL-HDD	96.02	95.44	95.80	95.62	2.18
HLDA-MALO	97.35	97.02	97.10	97.06	1.96
ML-CHDPM	98.12	97.84	97.91	97.88	1.71
XGBoost	98.46	98.10	98.24	98.17	1.58
HBW-SSG-CNN (Proposed)	99.21	98.94	99.08	99.02	1.31

The comparative findings 8 show that the proposed HBW-SSG-CNN system is consistently more effective than current machine learning and deep learning methods across all assessment measures. Combining optimized feature selection, attention-based parallel convolution, and Black-Winged Kite optimization significantly increases classification accuracy while reducing computational time. The increased values of sensitivity and specificity indicate strong disease detection and low false-positive rates. The results confirm the efficiency, scalability, and real-time appropriateness of the suggested IoMT-based heart disease monitoring system^41^.

### Statistical significance analysis

To rigorously demonstrate the reliability and statistical superiority of the HBW-SSG-CNN model, a comprehensive analysis of its results is conducted against a range of advanced models, including OA-CNN, SVM-IoMT, TabNet-CatBoost, IoTDL-HDD, HLDA-MALO, ML-CHDPM, and XGBoost. Several experimental sessions are conducted under the same training–testing conditions to enhances fairness. Hypothesis testing is conducted using performance indicators, including classification accuracy, sensitivity, specificity, and F1-score. Nonparametric and parametric statistical tests, such as the paired t-test, Wilcoxon signed-rank test, Friedman’s ranking test, and Nemenyi post hoc analysis, are used to determine whether observed improvements are statistically significant, consistent, and not random.

Table 9 summarizes a statistical comparison of all the evaluated models. Paired t-test results demonstrate that the comparison across all competing techniques yields p-values less than 0.05 relative to HBW-SSG-CNN, indicating a statistically significant performance improvement. Small confidence intervals indicate consistency in accuracy across repeated runs. The values of the Wilcoxon signed-rank test also confirm nonparametric significance. The proposed framework has the lowest mean rank (1.19) in the Friedman ranking, whereas the Nemenyi post-hoc test proves that it is better than all baselines. The confidence intervals are computed based on repeated experimental runs to capture variability in model performance^42^.

**Table 9:** Comprehensive statistical significance comparison.

Technique	p-value (t-test)	95% CI (Accuracy)	Wilcoxon p-value	Friedman mean rank	Nemenyi (vs Proposed)
OA-CNN	0.021	[89.68, 91.15]	0.0018	7.82	Significant
SVM-IoMT	0.015	[90.72, 92.01]	0.0012	6.94	Significant
TabNet-CatBoost	0.011	[91.55, 92.84]	0.0009	6.21	Significant
IoTDL-HDD	0.008	[92.41, 93.66]	0.0006	5.48	Significant
HLDA-MALO	0.007	[93.12, 94.29]	0.0005	4.76	Significant
ML-CHDPM	0.006	[93.54, 94.62]	0.0004	3.92	Significant
XGBoost	0.005	[94.09, 95.15]	0.0003	2.68	Significant
HBW-SSG-CNN (Proposed)	< 0.001	[98.90, 99.45]	< 0.001	1.19	Baseline

### Ablation study

A study of ablation is conducted to compare the individual and compound performance of the Hybrid Structural Graph Attention Network (HS-GAT), the Spherical Convolutional Neural Network (SCNN), and the Black-Winged Kite Algorithm (BKWA). All configurations are evaluated under the same experimental conditions to measure their effects on heart disease classification performance.

It is important to note that the ablation study results are obtained under controlled experimental conditions to evaluate the individual contribution of each module. These results do not represent the fully optimized performance of the proposed HBW-SSG-CNN model. The final performance metrics reported in Table 10 correspond to the complete model trained with full parameter optimization and tuning. The ablation findings 11 show the cumulative improvement in performance through the integration of each architectural element. The HS-GAT method is efficient at capturing inter-feature relationships, whereas the SCNN is effective at representing cardiac signals in both spatial and spectral domains. This combination (HS-GAT-SCNN) is significantly more productive across all metrics, indicating that it learns features that are complementary. The BKWA addition further streamlines network parameter selection and feature selection, resulting in the best accuracy (96.34) and F1-score (96.07). These results support the idea that each module plays a significant role in the strength and efficiency of the proposed framework. The ablation experiments are conducted under reduced training configurations to isolate the effect of individual components and therefore may not reflect the peak performance of the fully optimized model.

**Table 10:** Ablation study of the proposed HBW-SSG-CNN framework under controlled experimental settings.

Model configuration	Accuracy (%)	Sensitivity (%)	Specificity (%)	F1-Score (%)
HS-GAT	93.14	92.76	93.01	92.89
SCNN	93.68	93.22	93.54	93.31
HS-GAT-SCNN	95.02	94.71	94.88	94.79
HS-GAT-SCNN-BKWA (Proposed)	96.34	96.01	96.18	96.07

### Discussion

The experimental findings, in their entirety, demonstrate the efficiency and stability of the proposed IoMT and Blockchain-Based Heart Disease Monitoring System using HBW-SSG-CNN. The computation of the simulation parameters in Table 2 validates that the analysis is carried out under realistic IoMT conditions, using benchmark datasets, with the correct signal durations, and with equal training and testing fractions, which supports real-time heart disease monitoring the reliability of the experiment. The dataset description demonstrates the variety of ECG, PCG, and clinical features, supporting the generalizability of the proposed framework. The sample results from the different preprocessing phases show significant noise reduction, baseline correction, and improved time–frequency representations, providing a solid basis for accurate feature extraction.

Figure 4 shows that the training and test curves converge quickly with minimal overfitting, confirming that the trained models exhibit stable learning behavior. This ROC analysis in Fig. 5 once again demonstrates the high discriminative ability, and the proposed model has an AUC of 0.997. The ability to maintain constant error-rate stability between epochs in Fig. 6 indicates stability to training variability. Assessments involving blockchain indicate the effectiveness of proposed hashing and consensus functionality. The decrease in hash generation latency is shown in Fig. 7, whereas the faster and more consistent optimization convergence is demonstrated in Fig. 8 relative to baseline algorithms.

The comparative results in Tables 4 and 8 clearly indicate that HBW-SSG-CNN outperforms the existing methods across all classification measures, achieving higher accuracy, sensitivity, and specificity, and lower computation time. Tables 5 and 6 confirm the efficiency of blockchain in terms of security, latency, throughput, and scalability. The results of statistical significance in Table 8 show that the gains are not due to chance. Finally, the ablation experiment in Table 9 shows that the combination of HS-GAT, SCNN, and BKWA as a whole can improve the performance of the proposed framework. The use of cross-validation and consistent performance across folds indicates that the proposed model does not suffer from significant overfitting despite its architectural complexity. It is important to note that the blockchain performance evaluation is conducted in a simulated Python environment and does not represent deployment on a real distributed blockchain network. Factors such as network latency, node heterogeneity, and real-world adversarial conditions are not fully captured in this setup. Although the proposed framework demonstrates improved efficiency and reduced latency, security properties such as resistance to adversarial attacks (e.g., Sybil attacks, 51% attacks) are not explicitly evaluated and remain part of future work. In this study, real-time monitoring refers to near real-time processing with sub-second to few-second latency, which is suitable for continuous cardiac monitoring scenarios. Although the proposed model demonstrates high classification accuracy, interpretability remains an important consideration for clinical adoption. The hybrid graph attention mechanism inherently provides a level of interpretability by assigning attention weights to feature relationships, indicating their relative importance in decision-making. Future work will focus on incorporating explainable AI techniques to provide transparent and clinically interpretable insights, thereby improving trust and usability in real-world healthcare applications. The overall system latency includes signal acquisition, preprocessing, classification, and blockchain transaction time, resulting in an approximate end-to-end delay of around 1.5–2 s. While effective for continuous monitoring, further latency reduction may be required for time-critical emergency conditions.

## Conclusion

This study introduced a sophisticated heart disease monitoring IoMT and blockchain-based technology based on deep learning, tested and proven through simulation in Python. The suggested framework merges ECG and PCG acquisition using IoMT, QCBF to improve signal quality, SE-AEFD to perform adaptive decomposition, ST-QQPFT, and SBOA to reduce dimensionality, enabling reliable, real-time cardiac monitoring. To perform intelligent diagnosis, the HS-GAT-SCNN model is effective for modelling complex structural and spherical feature relationships, whereas BKWA is effective for optimizing the model’s parameters. The integrity of the data and assured sharing are enhanced by AHA-WPM hashing and FCB-HM consensus.

The results of the experiment show better performance with 99.21% accuracy, 98.94% sensitivity, 99.08% specificity, and 99.02% F1-score, and a shorter computation time of 1.31 s. A good discriminative capability is confirmed by ROC analysis, with an AUC of 0.997, and convergence graphs demonstrate stable learning and low error rates. The suggested framework addresses the significant issues identified in the problem statement, including noise signal acquisition, redundant high-dimensional features, low classification capability, blockchain latency, and high energy usage.

Although highly effective, this framework has limitations, including increased architectural complexity, higher memory requirements to model large-scale graphs, and reliance on optimal sensor calibration. Further development should focus on lightweight edge-aware deployment, adaptive graph scarification, integration of federated learning to preserve privacy, and real-world clinical validation across varied populations. These improvements will provide additional scalability, interpretability, and deployment usability for next-generation smart healthcare systems. Although the proposed framework demonstrates strong performance, several directions for future research remain. Future work will focus on lightweight and edge-aware implementations to reduce computational overhead and enable real-time deployment on resource-constrained IoMT devices. Additionally, integrating federated learning techniques can enhance data privacy by enabling decentralized model training. Further research will also explore advanced explainable AI methods to improve interpretability and clinical trust. Finally, real-world clinical validation across diverse patient populations and deployment on actual blockchain networks will be investigated to further validate the practicality and scalability of the proposed system.

## Data Availability

The datasets used and/or analysed during the current study available from the public repository https://physionet.org and https://archive.ics.uci.edu
